# The Equine Temporomandibular Joint: Comparisons Between Standard and Needle Arthroscopic Examination of Cadaver Specimens and Standing Horses

**DOI:** 10.3389/fvets.2022.876041

**Published:** 2022-04-26

**Authors:** James L. Carmalt, Karen L. Pimentel

**Affiliations:** Department of Large Animal Clinical Sciences, Western College of Veterinary Medicine, University of Saskatchewan, Saskatoon, SK, Canada

**Keywords:** arthroscopy, temporomandibular joint, fiberoptic, needle arthroscopy, video arthroscopy

## Abstract

**Background:**

Definitive diagnosis of equine temporomandibular joint osteoarthritis (TMJ-OA) may require advanced diagnostic imaging. Arthroscopy is a modern, minimally invasive, diagnostic, and treatment modality. Standing arthroscopic treatment of joint disease is a relatively recent advance in equine surgery, despite which there are few published comparisons between the available arthroscopic systems.

**Objective:**

To compare and contrast two arthroscopic systems for assessing the equine temporomandibular joint compartments in cadavers and standing horses.

**Study design:**

Experimental study.

**Methods:**

Phase I involved the assessment of the discotemporal joint (DTJ) and discomandibular (DMJ) joint compartments of both temporomandibular joints (TMJ) of 14 cadaveric equine heads using a caudally placed arthroscopy portal. Joints were initially examined using the needle arthroscope and the results compared to the findings of examination using a 2.5 mm 30° arthroscope system (standard). Three healthy horses were subsequently examined to determine the validity of the procedure in live animals in Phase II.

**Results:**

Needle and standard arthroscopy, in combination with mandibular manipulation, allowed evaluation of the caudal aspects of both joint compartments of the TMJ in Phase I. However, the extreme margins of the joint were more commonly visualized using standard arthroscopy. Live horses in phase II were restrained in stocks and both the rostral and caudal aspects of the DTJ and DMJ compartments of both TMJs were examined successfully understanding sedation and local analgesia. The use of a modified Guenther speculum allowed the mandible to be manipulated and offset, which facilitated a complete examination of the joint compartments. Despite adverse behavior encountered during the procedure in one horse, no surgical complications ensued.

**Main Limitations:**

Not blinded—bias; learning curve.

**Conclusions:**

The needle arthroscope system is a relatively inexpensive diagnostic tool, which can be used to evaluate the TMJ in the absence of advanced diagnostic imaging such as computed tomography or magnetic resonance imaging. However, if arthroscopic treatment is required after advanced imaging and pre-operative diagnosis, superior image quality and ease of manipulation may favor the use of the standard equipment.

## Introduction

Diseases of the temporomandibular joint (TMJ) are a common problem in humans and domestic animals. They encompass both intra- and extra-articular conditions which, in humans, accounts for significantly higher health care costs and sick leaves in affected vs. non-affected individuals (1).

Similar to the human anatomy, the equine TMJ is comprised of two independent synovial compartments (2), the larger dorsal discotemporal joint (DTJ) and the smaller discomandibular joint (DMJ), separated by a biconcave intra-articular disc. The arthroscopic approach and anatomy of the equine joint compartments have been well-described (3–7).

Osteoarthritis of the human TMJ (TMJ-OA) can be a primary condition or, less commonly, occurs secondary to trauma. Until recently, equine TMJ literature focused on TMJ-OA as a result of trauma and sepsis (8–16). However, over 35% of horses older than 1 year of age undergoing CT for reasons other than TMJ disease have been reported to have changes in their TMJs suggestive of osteoarthritis, despite not having any clinical signs attributable to disease of this joint (17). Equine TMJ-OA has also been associated with colic (18), poor performance (19), and behavioral abnormalities under saddle (20). Specialized radiographic projections have been described to evaluate this joint (21, 22); however advanced imaging, including contrast-enhanced computed tomography (CT), has been shown to be useful in the assessment of this joint (2). When access to these advanced modalities is limited, or when surgical treatment is required, arthroscopic techniques offer the ability to thoroughly assess the articular cartilage and intra-articular disc (3, 19, 23).

The objective of the study was to compare and contrast the use of a 65-mm-long, 1.2-mm-diameter, 10° forward viewing angle (FVA) needle arthroscope system (NAS), and a standard 2.5-mm-diameter, 30° FVA arthroscope (STAN) for the assessment of the equine TMJ. Further, to validate the technique and feasibility in living, standing, horses.

The hypotheses were that arthroscopy using NAS would be as effective in assessing the TMJ as the STAN method and that either technique would be successfully employed in standing horses.

## Materials and Methods

### Phase I—Cadaver Specimens

The study population comprised of 14 cadaver heads from 8 geldings and 6 mares with an age range of 2–30 years old (mean 17 years, SD ± 8.8) which had been euthanized for reasons unrelated to the study. As previously reported (7) heads were placed in lateral recumbency with a roll of towels under the opposite TMJ to allow angulation of the head in its long-axis toward the surgeon by ~30°. Using a full-mouth speculum (Stubbs Equine Innovations, Johnson City, TX, USA) the mandible of each head was opened maximally prior to surgical intervention to allow unimpeded manipulation during the procedure. The ipsilateral aural pinna was taped to the contralateral one to pull it caudally and away from the surgery site. The same surgeon performed all of the arthroscopies (cadaver and live horses) to ensure consistency.

To prevent fluid extravasation due to the differences in the size of the arthroscopic cannulae, the joint compartments of the TMJs were first examined using NAS and then STAN. Each TMJ was palpated identifying the intra-articular disc and caudal extent of the condylar process of the mandible. To assess the DTJ a 20G 1″ needle was placed directly into the joint compartment in a lateromedial direction immediately dorsal to the intra-articular disc and ventral to the lateral extent of the mandibular fossa of the temporal bone. Access to the DMJ was achieved by placing the 20G 1″ needle in either a lateromedial direction, dorsal to the condylar process of the mandible, immediately under the intra-articular disc; or as previously described, into the caudal aspect of the joint from a caudodorsal to the rostroventral direction (when viewed from the side of the horse) (7). Irrespective of needle position, ventral deflection of the hub resulting in dorsal movement of the intra-articular disc was used to confirm placement. Five milliliters of saline were used to distend the joint compartments. DTJ access was confirmed by distention of the joint capsule dorsal to the intra-articular disc with retention of the anatomical contour of the caudal aspect of the mandible. DMJ access was confirmed when the joint compartment at, and below, the palpable level of the condylar process of the mandible occurred. Additionally, rostral and lateral distention of the joint under the intra-articular disc could be palpated. Fluid reflux into the syringe when the plunger pressure was released was another way to confirm correct needle placement into either joint space. Irrespective of the chosen joint compartment, or arthroscope system, a straight Beaver (376700) blade was used to make a vertical skin and capsular incision.

A wide-bore (high flow) cannula and blunt obturator were inserted through the incision into the joint when using NAS. Once within the joint, the obturator was replaced with a 1.2 mm-diameter, 10° forward-viewing needle arthroscope (Biovision, Denver, USA). A fluid line attached to an arthroscopic fluid pump (SCB Hamou Endomat 263310 20, Karl Storz Endoskope, Tuttlingen, Germany) was used to maintain the intra-articular pressure at between 110 and 150 mm Hg. This pressure was consistent between arthroscope systems. Confirmation of entry into the correct joint compartment was determined when the dorsal aspect of the compartment was noted to be the articular cartilage of the mandibular fossa (DTJ); or the intra-articular disc (DMJ) which billowed synchronously with the incoming fluid. Additionally, in the latter scenario, the linear rostrocaudal striations of the fibrocartilage covering the condylar process of the mandible could be identified ventrally.

Joint exploration subsequently followed a routine pattern to identify specific anatomical structures within the joint compartment. Data were recorded as seen or not seen (Table 1). Subsequent to thorough joint exploration the NAS was removed and replaced with the STAN system 3.5 mm arthroscopy sheath. The obturator was removed and a 2.5 mm 30° arthroscope (ConMed Linvatec, Largo, FL, USA) was inserted into the joint. The same routine pattern of joint exploration was repeated with this arthroscopic system and the findings were recorded as described above.

**Table 1:** Anatomical structures are visualized with each of the arthroscope systems.

**Compartment**	**Anatomical Structure**	Visualized^*^		***p*-value**
		**STAN**	**NAS**	
Dorsal	Medial aspect of joint	26	18	0.02
Dorsal	Caudal aspect of joint	26	22	0.25
Dorsal	Articular eminence	28	26	0.49
Dorsal	Mandibular fossa of temporal bone	28	26	0.49
Dorsal	Intra-articular disc	28	26	0.49
Dorsal	Retro-discal tissue	25	18	0.06
Dorsal	Rostral attachment of disc	14	1	<0.001
Dorsal	Lateral attachment of disc	6	2	0.25
Dorsal	Rostral aspect of joint	18	6	0.003
Ventral	Medial aspect of joint	27	13	<0.001
Ventral	Mandibular condyle	28	25	0.24
Ventral	Intra-articular disc	28	25	0.24
Ventral	Caudal synovial plicae	26	21	0.14
Ventral	Lateral attachment of disc	20	6	<0.001
Ventral	Lateral cul-de-sac	19	9	0.02
Ventral	Rostral aspect of joint	15	5	0.01
Ventral	Retro-discal tissue	9	1	0.01

* *Visualized numbers represent the sum of both (the left and right) TMJs for each of the 14 horses, for a maximum total of 28*.

### Phase II—Live Animals

Three horses donated for teaching advanced techniques, approved by the institutional Animal Research Ethics Board, with no apparent dental or TMJ disease, were used in this portion of the study. Horses were restrained in stocks and sedated using 2 mg detomidine hydrochloride and 2 mg butorphanol tartrate through an intravenous catheter placed in the left jugular vein. Top-up sedation using the same dose of medication was administered as necessary. A modified Guenther full-mouth speculum (Equine Dental Instruments, Elmwood WI) was placed on the head, and the skin caudal to the eye and cranial to the ear was shaved and aseptically prepared in a routine manner for arthroscopic surgery. A large 3″ by 3″ square was desensitized using line blocks of 2% lidocaine, leaving the contours of the TMJ readily palpable in the center of the prepared area. Due to the size difference between the NAS and STAN systems, live horses were examined in the same order as that reported for Phase I. The condylar process of the mandible and intra-articular disc of the left TMJ were identified by palpation and a 22G 1″ needle was inserted into the caudal recess of the DMJ in a lateral to medial direction. Lack of resistance to fluid flow was taken as an indication of successful entry into the joint, after ventral deflection of the needle hub resulted in appreciable movement of the intra-articular disc, 4 ml 2% lidocaine was injected into the joint. The rostral recess was seen to distend laterally, with no obvious change in the contour of the tissues above the intra-articular disc. The needle was removed and entry into the joint was performed using a Beaver blade as previously described above. After placement of the NAS, the mandible of the horse was moved, by an assistant, away from the surgeon, and the bite plate was secured in position using the retaining pin. After joint exploration using NAS, the arthroscopic equipment was removed and the mandible returned to the neutral (midline) position before the STAN instrumentation was inserted into the joint and the mandibular excursion was repeated. After completing the examination of the DMJ, the DTJ was entered and explored using the same sequence of events.

### Data Analysis

A two-sided Fisher's Exact test was used to compare the ability of the two arthroscopic systems to visualize individual anatomical structures in the TMJ joint compartments. A chi-squared test was used to compare the overall ability of the two systems to identify anatomical structures of interest and to evaluate whether there was a joint compartment effect in this ability. *P*-values <0.05 were considered significant.

## Results

### Cadaver Specimens

**Figure 1:**
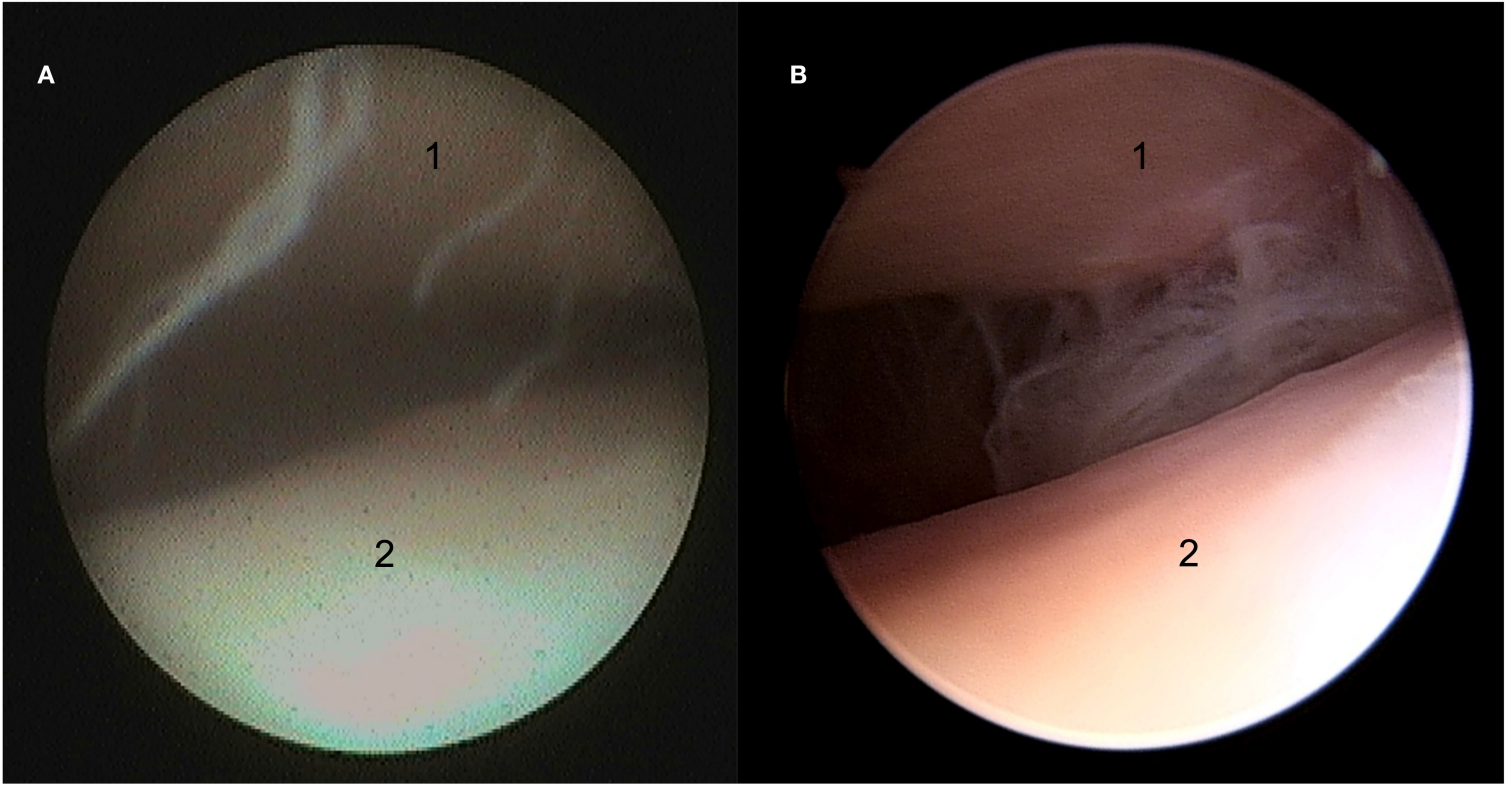
Still arthroscopic image of the medial aspect of the discotemporal joint (DTJ) using the needle (NAS) **(A)** and standard (STAN) **(B)** arthroscopic systems. 1, the articular eminence of the temporal bone; 2, intra-articular disc.

**Figure 2:**
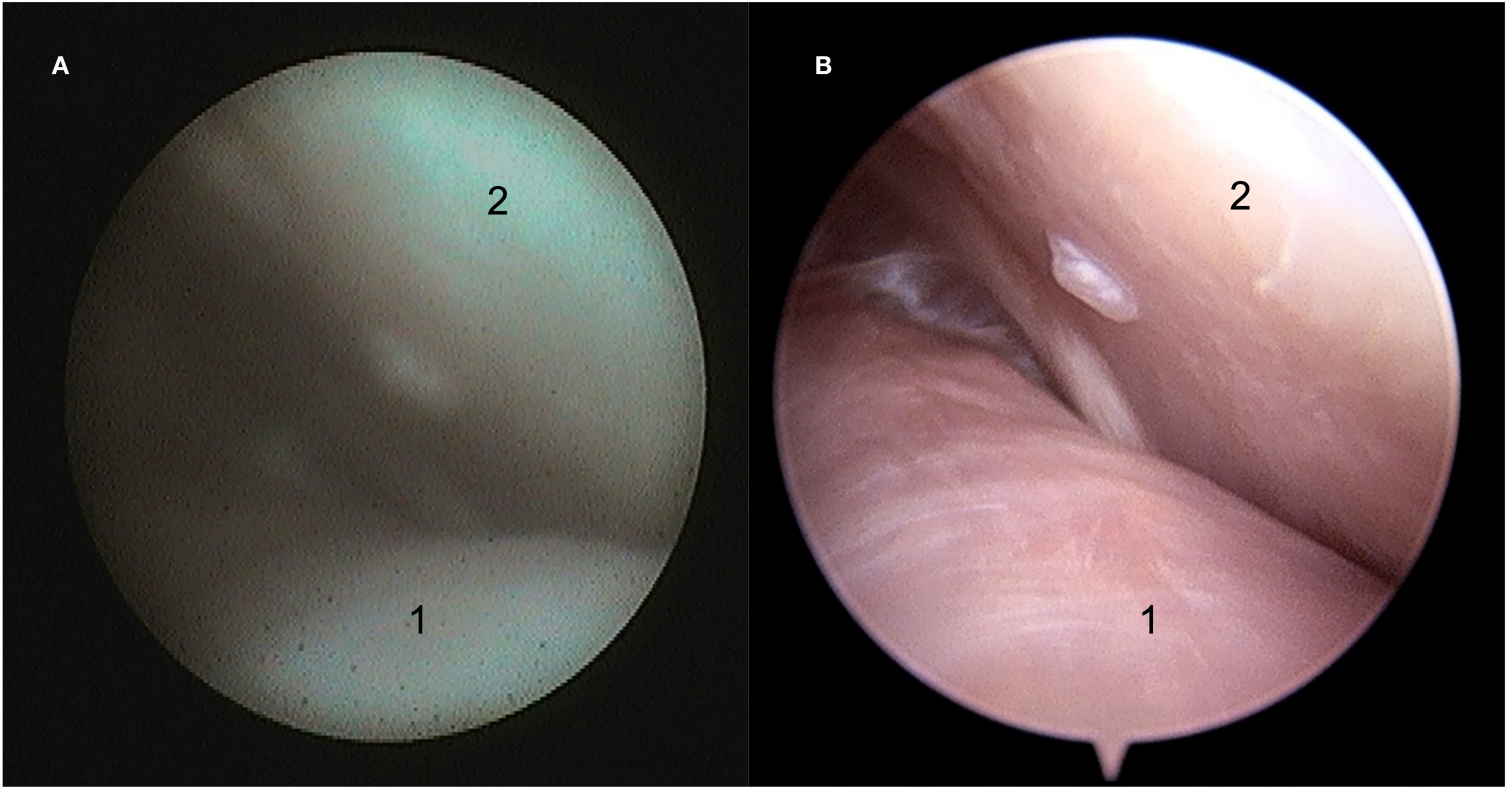
Still arthroscopic image of the caudolateral aspect of the discotemporal joint (DTJ) using the NAS **(A)** and the STAN **(B)**. 1, intra-articular disc; 2, caudal aspect of the joint.

**Figure 3:**
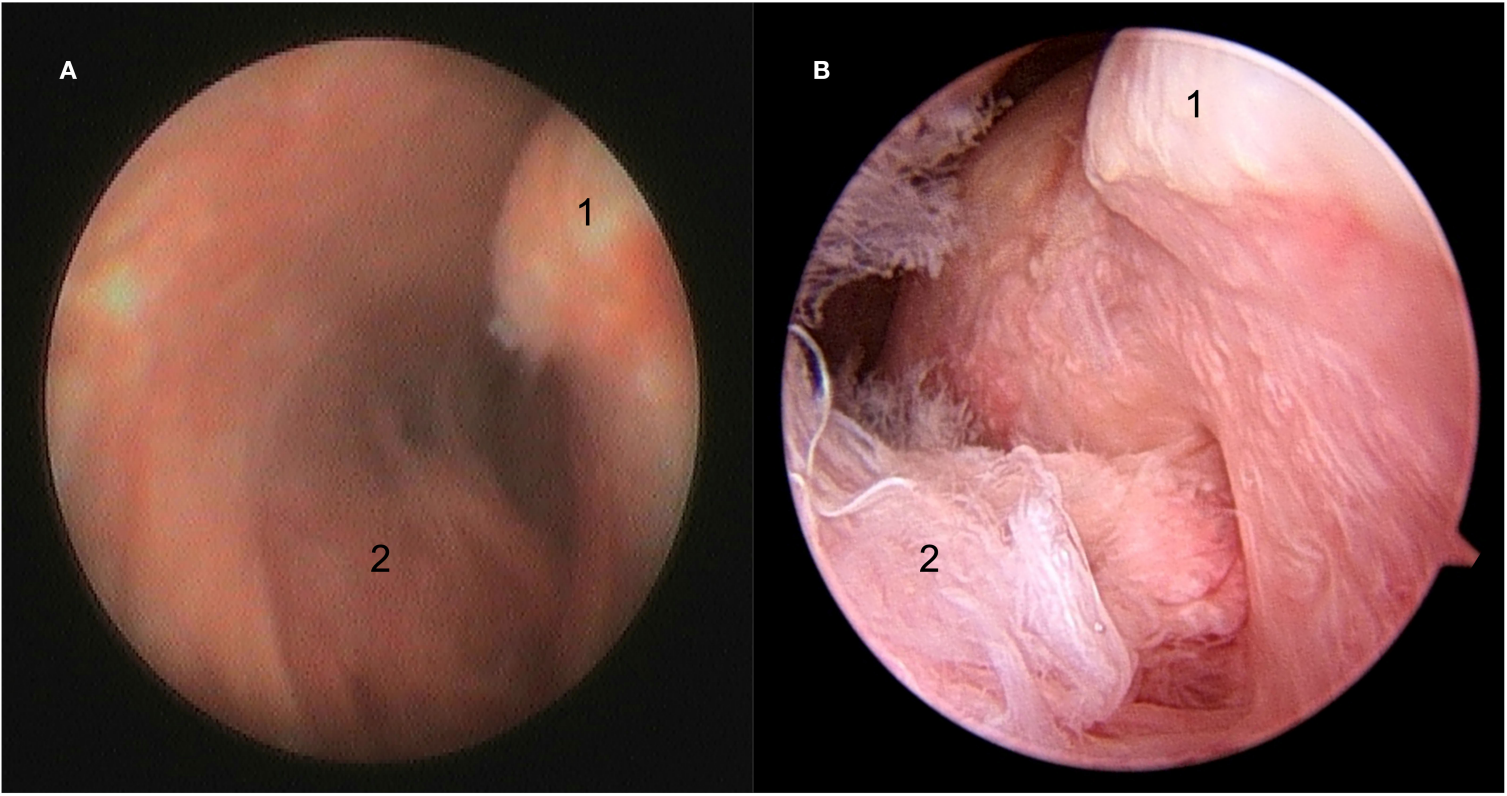
Still arthroscopic image showing the caudal aspect of the condylar process of the mandible (1) with synovial plical insertion (2) within the discomandibular joint (DMJ) using the NAS **(A)** and STAN **(B)**.

**Figure 4:**
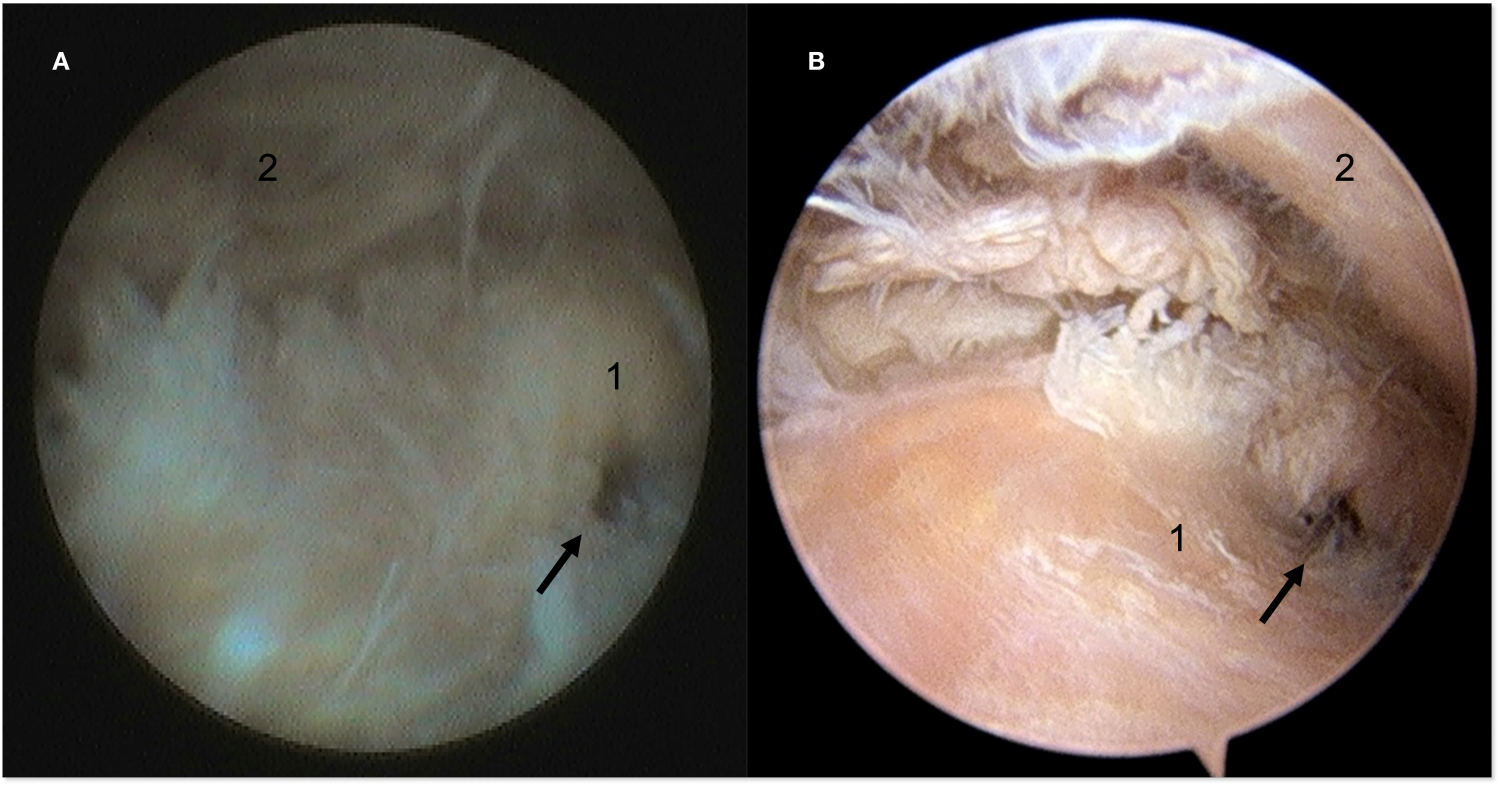
Still arthroscopic image of the caudomedial aspect of the DTJ using the NAS **(A)** and the STAN **(B)**. A perforation (black arrow) in the intra-articular disc was identified at the caudomedial fibrous expansion (cfe) with both modalities. 1, intra-articular disc; 2, articular eminence of the temporal bone.

**Figure 5:**
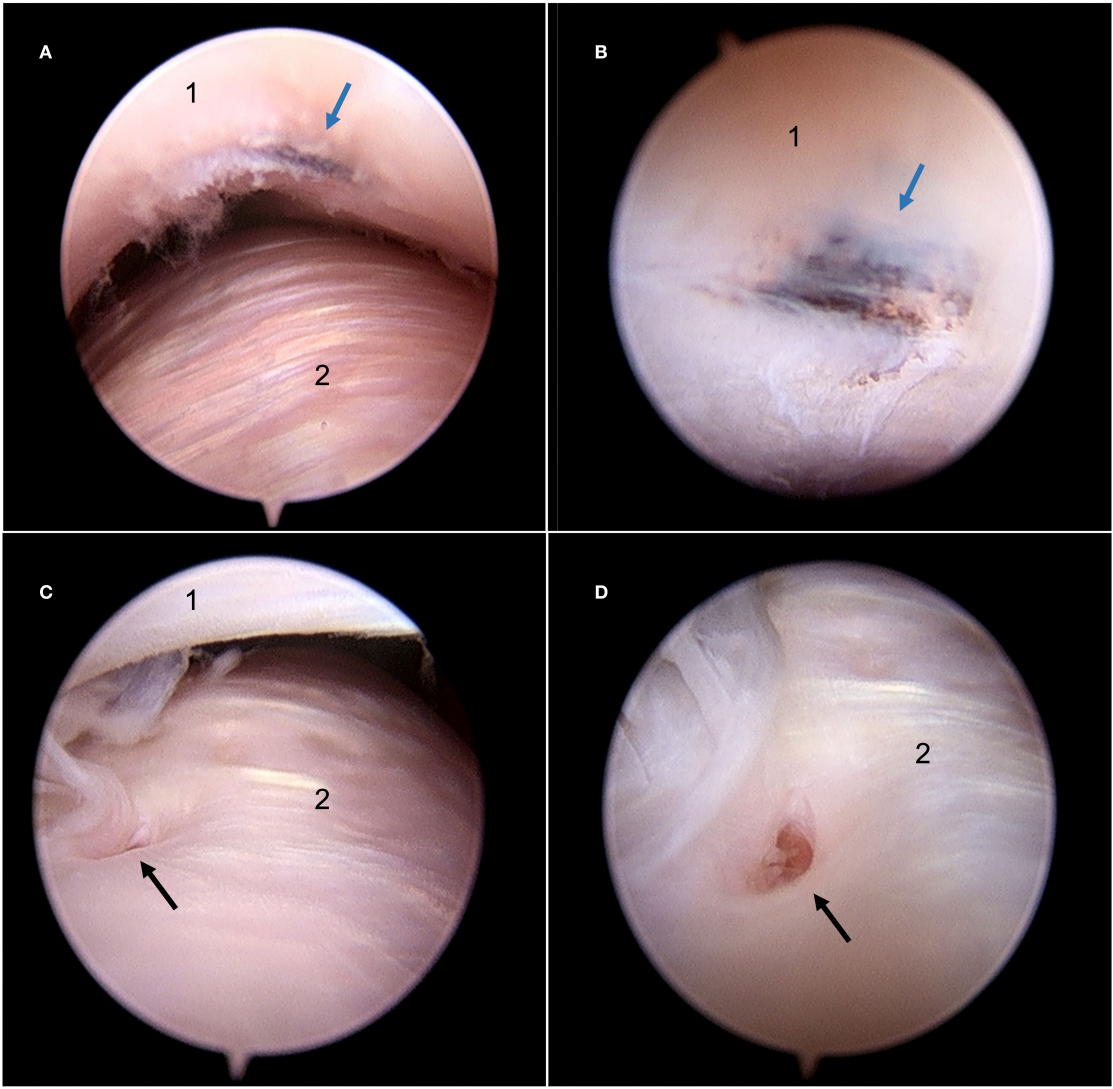
Still arthroscopic image of the DMJ using the STAN illustrating two abnormalities identified with this system which were not identified during joint exploration using the NAS. **(A)** View of an abnormal discoloration on the ventral aspect of the intra-articular disc from a distance. **(B)** Advancing the arthroscope highlights the dark pigmentation (blue arrow) present on the ventral aspect of the intra-articular disc. **(C)** Caudal aspect of the condylar process of the mandible with a defect on the articular surface. **(D)** Advancing the arthroscope confirms the presence of a cloaca associated with a mandibular cyst (black arrow). 1, Intra-articular disc; 2, condylar process of the mandible.

Complications entering the joint compartments were common in the first few cadaver specimens and hinged on adapting to the flexibility of the NAS instrumentation compared to the rigidity of the STAN systems. Additionally, the use of Beaver blades to create arthroscopic portals after joint distention was critical in preventing significant fluid extravasation as previously reported (7). Determining whether the correct compartment (DMJ or DTJ) had been entered was not immediately clear in the early phase of the cadaver study (Figure 1) as the investigators had to adapt to the image quality of the NAS (Figures 2, 3). Additionally, when attempting entry into the DMJ, the reported visual cue of billowing of the intra-articular disc was frequently absent when using this equipment. Multiple abnormalities were identified with both systems (Figure 4); however, some were missed during NAS exploration (Figure 5). As the operator became more familiar with the imaging of the NAS, these abnormalities were being more consistently identified.

### Live Animals

Arthroscopic evaluation of the joint compartments of both TMJs was successfully performed in all three live horses. Placement of the modified Guenther speculum, which allowed mandibular manipulation by an assistant, was integral to the successful evaluation of the joint compartments. It allowed mandibular distraction and prevented the horses from chewing, which causes cavitation of the intra-articular fluid and potential damage to the arthroscopic equipment and intra-articular structures. Working within the relatively large de-sensitized region allowed the surgeon to palpate the anatomically pertinent landmarks necessary for correct arthroscope placement—namely the external margins of the mandibular fossa of the temporal bone, and the condylar process of the mandible, as well as the intra-articular disc—prior to distending the joint with a local anesthetic solution. The ease of arthroscope placement would not have been possible if these structures had been obscured by placing local analgesia solution solely at the site of the projected arthroscopy portal. The sedative and local analgesic protocol worked well in two of the three horses, but one horse was unexpectedly responsive to touch and noise during the procedure. This horse would aggressively throw his head up, or rotate it, at times, which complicated joint exploration. Despite the need for repeated re-introduction of the arthroscope, completion of the procedure occurred, which was aided by having the arthroscopes held such that the middle finger of the surgeon's hand manipulating the instrument was in contact with the skin of the head (within the desensitized region). This allowed the surgeon to grasp the arthroscope securely when it was dislodged from the joint, while preventing it from being driven deeply, and uncontrollably, into the joint when the horse unexpectedly turned its head toward the surgeon.

### Statistical Analysis

A total of 14 horses, each with two TMJs, for a total number of 28 joints had nine anatomical structures of interest in each of the DTJs and eight in each DMJ compartment examined. Therefore, if all structures were visualized in all DTJs, then 252 would have been identified. Similarly, if this occurred in the DMJs, then 224 would have been identified. Both the NAS and STAN equipment allowed examination of the joint compartments of the TMJ, however adequate visualization of the most lateral and rostral aspects of the joint was more frequently compromised when using the NAS than the STAN systems (Table 1). Overall, significantly more anatomical structures were identified with the STAN system (*p* < 0.0001; Table 2). The ability of the STAN system to identify anatomical structures was independent of the joint compartment examined (*p* = 0.57) however this was not the case with the NAS (*p* = 0.02, Table 3). NAS allowed the identification of more structures in the DTJ, but less in the DMJ than expected.

**Table 2:** The total number of anatomical structures visualized compared by arthroscope system and joint compartment.

		**Number of**
		**anatomical**
		**structures**
**Compartment**	**System**	**visualized**	**%**	***p*-value**
Dorsal (*n* = 252)	STAN	199	79.0	<0.001
	NAS	145	57.5	
Ventral (*n* = 224)	STAN	172	76.8	<0.001
	NAS	105	46.9	

**Table 3:** A comparison between arthroscope systems by joint compartment.

		**Number of**
		**anatomical**
		**structures**
**System**	**Compartment**	**identified**	**%**	***p*-value**
STAN	Dorsal	199	79.0	0.57
	Ventral	172	76.8	
NAS	Dorsal	145	57.5	0.02
	Ventral	105	46.9	

## Discussion

The primary objective of the study was to compare and contrast the use of a 65-mm-long, 1.2-mm-diameter, 10° forward viewing angle (FVA) needle arthroscope (NAS), and a standard 2.5-mm-diameter, 30° FVA arthroscope (STAN) for the assessment of the equine TMJ.

While the use of both sets of equipment requires an intimate knowledge of anatomy and arthroscopic principles, the authors would suggest that most surgeons are more comfortable with, and have been trained in, the use of the STAN equipment. The NAS is fundamentally different in its design, being a fiberoptic endoscope rather than a video endoscope, and by being limited to a 10° FVA rather than a 30° FVA commonly used in the STAN arthroscopes. The majority of the intra-articular exploration reported in this study was unaffected by these limitations—beyond that of image quality. However, there were two situations in which the difference between the equipment was most appreciable. Firstly, when confirming entry into the DMJ surgeons have become accustomed to assessing the movement of the intra-articular disc. This is especially useful in low visibility conditions, such as in the presence of purulent material or blood. The inherent difference in image quality between the NAS and STAN hampers this assessment. Further, despite using the same pressure settings on the fluid pump, there is a fundamental limit on the flow rate of fluids into the joint compartment when using the NAS, even when the high-flow cannula was used. In most specimens, and in the live horses, this lack of flow prevented the use of intra-articular disc movement as confirmation of DMJ entry. Secondly, when attempting to view the most lateral aspect of the joints, portions of the rostral aspect of the joint compartment, and enter the lateral cul-de-sac ventral to the condylar process of the mandible, the difference between the 10° and 30° FVA became apparent (Figure 3), especially as the authors elected to only access the caudal aspect of the joint compartments in the cadaver heads. While the flexibility of the NAS has been reportedly useful in other joints, it was frustrating in the TMJ. The authors routinely use the rigidity of the STAN equipment to aid in maneuvering the arthroscope within this joint. Even with practice, and the use of the surgeon's second hand to steady and stiffen the shaft of the NAS, a few areas of the joint remained a challenge to image, especially if the arthroscope portal was placed slightly too ventral, or caudal, or if there was a significant synovial proliferation in the caudal region of the joint.

The secondary objective of the study was to validate the technique and assess the feasibility, of examination of the TMJ in living, standing, horses using these arthroscopic systems. Standing surgery of the equine TMJ has been reported (11), and routine standing arthroscopic surgery has been reported as an alternative to that performed under general anesthesia in the TMJ and fetlock (24, 25). The NAS was initially reported as a diagnostic imaging modality in the equine stifle (26). More recently, the feasibility of its use has been reported in the equine caudal cervical articular process, scapulohumeral, hock, carpus, and fetlock joints, as well as a method to examine the sinuses and tendon sheath (27–33). These procedures avoid the risks and costs of general anesthesia, as well as reportedly reduce complications such as bleeding in some specific situations. The authors found that both the NAS and STAN arthroscopic equipment was well-tolerated by the horses and rendered an acceptable assessment of the joint, with no intra-operative complications noted during the use of either system. The use of the modified Guenther full-mouth speculum prevented inadvertent jaw motion and allowed mandibular displacement which has been reported as critically important in assessing the DMJ in horses (7, 20). The lack of jaw motion is an important consideration in the protection of the surgical equipment, but at the same time the lack of versatility, and immediacy, of mandibular motion is also an impediment to the arthroscopic assessment of the TMJ compartments. It is possible that the flexibility of the NAS would become significantly more important if used without a speculum, or in situations (as experienced in Phase II) where a horse's behavior precludes safe, standing arthroscopic assessment of the TMJ using the STAN system. Additionally, the fact that the NAS is fiberoptic means that even if broken in the process, it will not leave glass fragments within the joint.

There is an inherent bias in this study, which is important to acknowledge. As noted, the difference in size between the equipment necessitated that the NAS was used before STAN. Thus, any questions as to joint compartment of entry, possible pathological findings, etc., had already been discussed and agreed upon before the STAN equipment was placed. This led the authors, who were already more comfortable with the latter equipment, to have this bias reinforced. With that understood, there is no doubt that fundamental, inherent, differences between the NAS and STAN render the image quality between the systems incomparable, and these same differences prevent the NAS from having a 30° FAV. Despite these differences, NAS is substantially cheaper than STAN and allows exploration of the equine TMJ.

## Conclusion

The exploration of the synovial structure is one component of joint surgery. Given that one is unlikely to surgically explore a supposedly normal joint, there has to be an expectation of treatment when one initiates surgery. In the author's experience, arthroscopic exploration of the equine TMJ typically occurs after exhaustive investigation and advanced diagnostic imaging. In these cases, the superior image quality and ease of manipulation may favor the use of the STAN equipment. It is possible that the NAS will render a diagnosis in the absence of advanced imaging, but in this hypothetical situation, treatment is still likely to remain a necessity. As such, it is the opinion of the authors that the image quality of a small 30° FAV video endoscopic STAN system will be preferable to most surgeons.

## Data Availability Statement

The original contributions presented in the study are included in the article/supplementary material, further inquiries can be directed to the corresponding author/s.

## Ethics Statement

The animal study was reviewed and approved by University of Saskatchewan's Animal Research Ethics Board and adhering to the Canadian Council on Animal Care guidelines for humane animal use.

## Author Contributions

JC contributed to study concept, design and execution, data interpretation, and manuscript preparation. KP contributed to study design, data collection, interpretation and analysis, and manuscript preparation. Both authors approved of the final manuscript.

## Funding

This research was supported by a University of Saskatchewan—Accountable Professional Expense Fund and the needle arthroscopes were supplied by the manufacturer. The funding and equipment, sources had no role in study design, specimen collection, analysis, and interpretation of data, in the writing of the manuscript, or in the decision to submit the manuscript for publication.

## Conflict of Interest

The authors declare that the research was conducted in the absence of any commercial or financial relationships that could be construed as a potential conflict of interest.

## Publisher's Note

All claims expressed in this article are solely those of the authors and do not necessarily represent those of their affiliated organizations, or those of the publisher, the editors and the reviewers. Any product that may be evaluated in this article, or claim that may be made by its manufacturer, is not guaranteed or endorsed by the publisher.

## References

[1] Hoffmann RG Kotchen JM Kotchen TA Cowley T Dasgupta JM Cowley AW. Temporomandibular disorders and associated clinical comorbidities. Clin J Pain. (2011) 27:268–74. 10.1097/AJP.0b013e31820215f5 21178593

[2] Pimentel KL Carmalt JL. The frequency of communication between the synovial compartments of the equine temporomandibular joint: a contrast-enhanced computed tomographic assessment. Front Vet Sci. (2021) 8:753983. 10.3389/fvets.2021.753983 34760960 PMC8573115

[3] May KA Moll HD Howard RD Pleasant RS Gregg JM. Arthroscopic anatomy of the equine temporomandibular joint. Vet Surg. (2001) 30:564–571. 10.1053/jvet.2001.28438 11704953

[4] Weller R Maierl J Bowen IM May SA Liebich HG. The arthroscopic approach and intra-articular anatomy of the equine temporomandibular joint. Equine Vet J. (2002) 34:421–4. 10.2746/042516402776249155 12117118

[5] Stadtbäumer G Boening KJ. Diagnostische und arthroskopische Verfahren am Kiefergelenk des Pferdes. Tierärztl Prax. (2002) 30:99–106. 10.2106/JBJS.I.00667 21266638 PMC3028452

[6] McIlwraith CW Nixon AJ Wright IM Boening KJ. Arthroscopy of the temporomandibular joint. In: McIlwraith CW, Nixon AJ, Wright IM, Boening KJ, editors. Diagnostic and Surgical Arthroscopy of the Horse. 3rd ed. St. Louis, MO: Elsevier (2005). p. 441–5. 10.1016/B978-0-7234-3281-4.50019-1

[7] Carmalt JL Tucker ML. Arthroscopic approach and intra-articular anatomy of the equine discomandibular joint compartment of the temporomandibular joint. Vet Surg. (2020) 49:1326–33. 10.1111/vsu.13487 32633420

[8] Carmalt JL Wilson DG. Arthroscopic treatment of temporomandibular joint sepsis in a horse. Vet Surg. (2005) 34:55–8. 10.1111/j.1532-950x.2005.00010.x 15720597

[9] Frietman SK van Proosdij ER Veraa S de Heer N Ter Braake F. A minimally invasive partial condylectomy and temporal bone resection for the treatment of a suspected chronic synovial sepsis of the temporomandibular joint in a 3.5-year-old paint horse gelding. Vet Q. (2018) 38:118–24. 10.1080/01652176.2018.1535216 30773124 PMC6830993

[10] Carmalt JL. Temporomandibular joint disorders. In: Auer JA, Stick JA, Kümmerle J, Prange T, editors, *Equine Surgery*. 5th ed. St. Louis, MO: Elsevier (2018). p. 1789–93. 10.1016/B978-0-323-48420-6.00103-4

[11] Sanders RE Schumacher J Brama PAJ Zarelli M Kearney CM. Mandibular condylectomy in a standing horse for treatment for osteoarthritis of the temporomandibular joint. Equine Vet Educ. (2014) 26:624–28. 10.1111/eve.12235

[12] Balducci J Ruby J Hall C Williams J. Arthrotomy, curettage and medical management of septic arthritis and osteomyelitis of the temporomandibular joint in a horse. Equine Vet Educ. (2021) 33:5–11. 10.1111/eve.13156

[13] Barnett TP Powell SE Head MJ Marr CM Steven WN Payne RJ. Partial mandibular condylectomy and temporal bone resection for chronic, destructive, septic arthritis of the temporomandibular joint in a horse. Equine Vet Educ. (2014) 26:59–63. 10.1111/eve.12053

[14] Nagy AD Simhofer H. Mandibular condylectomy and meniscectomy for the treatment of septic temporomandibular joint arthritis in a horse. Vet Surg. (2006) 35:663–8. 10.1111/j.1532-950X.2006.00205.x 17026552

[15] Devine DV Moll HD Bahr RJ. Fracture, luxation, and chronic septic arthritis of the temporomandibular joint in a juvenile horse. J Vet Dent. (2005) 22:96–9. 10.1177/089875640502200204 16149388

[16] Warmerdam EP Klein WR van Herpen BP. Infectious temporomandibular joint disease in the horse: computed tomographic diagnosis and treatment of two cases. Vet Rec. (1997) 141:172–4. 10.1136/vr.141.7.172 9290196

[17] Carmalt JL Kneissl S Rawlinson JE Zwick T Zekas L Ohlerth S Bienert-Zeit A. Computed tomographic appearance of the temporomandibular joint in 1018 asymptomatic horses: a multi-institution study. Vet Radiol Ultrasound. (2016) 57:237–45. 10.1111/vru.12334 26773281

[18] Smyth T Allen AL Carmalt JL. Clinically significant, nontraumatic, degenerative joint disease of the temporomandibular joints in a horse. Equine Vet Educ. (2017) 29:72–7. 10.1111/eve.12382

[19] Jørgensen E Christophersen MT Kristoffersen M Puchalski S Verwilghen D. Does temporomandibular joint pathology affect performance in an equine athlete? Equine Vet Educ. (2015) 27:126–30. 10.1111/eve.12268

[20] Carmalt JL Reisbig NA. Arthroscopic treatment of bilateral mandibular condylar cysts and associated osteoarthritis of the temporomandibular joints in a horse. Equine Vet Educ. (2021) 10.1111/eve.13602. [Epub ahead of print].

[21] Ebling AJ McKnight AL Seiler GS Kircher P. A complementary radiographic projection of the equine temporomandibular joint. Vet Radiol Ultrasound. (2009) 50:385–91. 10.1111/j.1740-8261.2009.01554.x 19697603

[22] Townsend N Cotton J Barakzai S. A tangential radiographic projection for investigation of the equine temporomandibular joint. Vet Surg. (2009) 38:601–6. 10.1111/j.1532-950X.2009.00536.x 19573061

[23] Adams K Schulz-Kornas E Arzi B Failing K Vogelsberg J Staszyk C. Functional anatomy of the equine temporomandibular joint: histological characteristics of the articular surfaces and underlining tissues. Vet J. (2018) 239:35–41. 10.1016/j.tvjl.2018.08.003 30197107

[24] Elzer EJ Wulster KB Richardso DW Ortved KF. Standing arthroscopic treatment of temporomandibular joint sepsis in a horse. J Vet Dent. (2020) 37:94–9. 10.1177/0898756420948269 32815477

[25] Gasiorowski JC Richardson DW. Diagnostic and therapeutic arthroscopy in the standing horse. Vet Clin North Am Equine Pract. (2014) 30:211–20. 10.1016/j.cveq.2013.11.011 24680213

[26] Frisbie DD Barrett MF McIlwraith CW Ullmer J. Diagnostic stifle joint arthroscopy using a needle arthroscope in standing horses. Vet Surg. (2014) 43:12–8. 10.1111/j.1532-950X.2013.12068.x 24175893

[27] Pérez-Nogués M Vaughan B Phillips KL Galuppo LD. Evalution of the caudal cervical articular process joints by using a needle arthroscope in standing horses. Vet Surg. (2020) 49:463–71. 10.1111/vsu.13388 32022955

[28] Pouyet M Bonilla AG. Diagnostic needle arthroscopy of the scapulohumeral joint in standing sedated horses. Vet Surg. (2021) 50:29–37. 10.1111/vsu.13529 33074573

[29] Kadic DTN Bonilla AG. Diagnostic needle arthroscopy of the tarsocrural joint in standing sedated horses. Vet Surg. (2020) 49:445–54. 10.1111/vsu.13375 31943288

[30] Miagkoff L Bonilla AG. Diagnostic tenoscopy of the carpal sheath with a needle arthroscope in standing sedated horses. Vet Surg. (2020) 49:O38–44. 10.1111/vsu.13381 31981365

[31] Kadic DTN Miagkoff L Bonilla AG. Needle arthroscopy of the radiocarpal and middle carpal joints in standing sedated horses. Vet Surg. (2020) 49:894–904. 10.1111/vsu.13430 32333682

[32] Pouyet M Bonilla AG. Validation of a 2-mm videoendoscope for the evaluation of the paranasal sinuses with a minimally invasive technique. Vet Surg. (2020) 49:O60–70. 10.1111/vsu.13269 31228274

[33] Bonilla AG. Standing needle arthroscopy of the metacarpophalangeal and metatarsophalangeal joint for removal of dorsal osteochondral fragmentation in 21 horses. Vet Comp Orthop Traumatol. (2019) 32:420–6. 10.1055/s-0039-1688984 31127597

